# Environmental performativity: How natures are made

**DOI:** 10.1177/27539687251321503

**Published:** 2025-03-03

**Authors:** George Cusworth, Theo Stanley

**Affiliations:** Department of Philosophy, Classics, History of Art and Ideas, Georg Morgenstiernes Hus, Oslo, Norway; School of Geography and the Environment, Oxford, UK

**Keywords:** Performativity, environmental governance, environmental metrics, ecosystem services, neoliberal natures, reflexive performativity

## Abstract

A range of things get made as natures are subject to programmes of management, measurement, regulation, subsidy, and commodification. Knowledge production practices stabilise dynamic environments into governable objects. Environmental management schemes materialise specific ecologies in particular places and create value for certain actors. Neoliberal governance mechanisms reproduce their own ideological foundations. This paper offers a conceptual vocabulary to articulate the different performances and performative processes that drive these makings. Drawn together under the framework of environmental performativity, we show how *objects*, *environments*, and *regimes* all get made as natures are governed. Together, these terms disambiguate the diverging ways the concept of performativity gets used in the literature. They reveal how material, semiotic, ideological, and calculative forces exert their influence at different points in the design and delivery of environmental governance interventions. In describing these terms, we want to highlight the unique influence that human activities have in shaping the world without denying the agency of nonhuman actors and materialities. By drawing attention to the fact that alternative performances have the power to yield more just and sustainable environmental futures, we conclude the paper on a more optimistic note. We use the concept of reflexive performativity to discuss these political opportunities.

Fences make governable forests (Lansing 2012). Speculative proposals make energy transitions (Gabrys 2014). Imprecise measurements make viable emissions markets (Ghosh 2019). Across these studies, scholars have invoked the concept of performativity to account for the way material reality, semiotic representation, and knowledge production converge in programmes of environmental governance. In doing so, they have shown how nature is constantly (re)made as it is subject to diverse acts of measurement, management, and control.

As environmental geographers, political ecologists, and Science and Technology (STS) scholars continue to expand the list of actors and technologies that do ‘performative work’ (Beunza and Ferraro 2019), they are extending Michel Callon's (1998b) conclusion that *economists help form the economic reality they describe*. They have depicted the performances of field scientists and data managers that stabilise dynamic ecologies into enumerable and governable objects (Lansing 2012; Lippert 2015). They have shown how the metrics used to capture environmental data determine which natures get materialised through regimes of commodification (Palmer 2021). And they have shown how political ideals define how environmental problems are understood and how solutions are designed (Kolinjivadi et al. 2019; Turnhout, Neves and de Lijster 2014).

Whilst this literature has sought out the individual connections between ecological change, acts of scientific representation, and political ideology, the full set of causal connections this literature speaks to remain implicit. We read across this work to break down each step in the cycle, showing how regimes make environments, how representations make regimes, how environments are stabilised into governable forms, and how different performative makings iteratively condition one another. Cleaning up the conceptual slippage around the concept of performativity and synthesising the insights it has been used to generate is the main aim of the paper.

Building on pre-existing literature on the place of performativity in human geography (Dewsbury 2000) and environmental politics (Kolinjivadi et al. 2019), we suggest that one or more of three processes – each of which gets described in the literature as being performative – might be underway. These processes – *object making*, *environment making*, and *regime making* – constitute the overarching category of environmental performativity. Taken in aggregate, environmental performativity offers a framework for understanding how diverse material, calculative, and semiotic forces interact in environmental governance contexts. It accounts for the performative power of matter (Barad 2007) and the material entanglement of humans and nature (Latour 2005; Lorimer 2012), without relativising the unique power human representations and activities exert over the world (Lemke 2021).

*Object making performativity* refers to the way that various actors (scientists, foresters, ecologists etc.) tooled with material semiotic props (quadrats, spreadsheets, databases etc.) abstract messy ecological systems into discrete and enumerable units. The repetition of their performances bounds ontological categories and counts the objects that fit into them. *Environment making performativity* refers to the landscapes and ecological dynamics that get produced through diverse programmes of environmental governance. Here, the peculiarities of the object making performance are inscribed into the environments they help create. Shot through with justice-relevant outcomes, these specifics determine *which* natures are made, *where,* and *who benefits*. The cyclical object and environment making processes have performative outcomes of their own, stabilising the ontological, political, and epistemic regimes which informed their creation. We term this the *regime making performativity* of environmental governance. In the contemporary political context, in which neoliberal forms of environmental management predominate, the regime being enacted entails the continued de-politicization of environmental management, the sedimentation of functional human–nature relations, and the privileging of technocratic forms of knowledge production. Across the three constituent elements of environmental performativity, we tease apart the performances of different human, nonhuman, discursive, calculative, and technological actants from the performative outcomes they generate.

These disambiguated analytics help make clear how specific actors, processes, ideologies, technologies, and materialities exert influence at different points in the design and execution of diverse programmes of environmental governance. In disentangling these performances and performative outcomes, we also highlight the contingent ways that natures get known and acted on in political life. This contingency lies at the heart of the concept of *reflexive performativity* – a term we call on in the paper's conclusion to emphasise how different performances can be designed to yield more just and sustainable futures. Where vagueness has dulled the blade of criticism, we argue that a more clearly demarcated set of analytics can offer scholars the interpretive frames needed to critique the performance as well as the Promethean tools needed to re-write the script. Highlighting the emancipatory potential of the notion of performativity is the second aim of the paper.

Over the course of the review, we investigate the performativity of schemes which *make natures*. The term is deliberately ecumenical about the diverse forms of environmental governance (subsidies, markets, regulations, investment initiatives, taxation etc.) we include in our review. *Making natures* is a play on words. It relates to the interventions that make (build, create, put together) more of whatever ecology has been identified as desirable. It also refers to those interventions in which natures are being made (forced, coerced, compelled) to do something (e.g. reduce flood risk, provide ecosystem services). The phrase *making natures* also nods to the fact that the natures that get governed, traded, remunerated, or subsidised are, at least in part, representations that are performatively *made up* (fabricated, contrived, conjured)*.* We use the word *natures* to foreground the multiple and contingent trajectories along which ecologies can develop and the multiple ontologies that permit what ‘naturalness’ can mean (Lorimer 2012).

As part of the paper's contribution, we offer an exploded diagram of the *environmental performativity* concept, and the *object, environment,* and *regime* making components it is made up of. We refer back to the diagram over the course of the paper.

In the next section, we offer a brief introduction to the development of the notion of performativity as a way of situating our environmental performativity concept in its wider intellectual history. The subsequent three sections form the main substance of the review: the object making, the environment making, and regime making performativity involved in making natures. In the fifth and final section, we offer an agenda for a reflexive performativity. This amounts to a programme of research and action that uses the (newly disambiguated) concept of environmental performativity to both read and write for difference in the pursuit of more just environmental futures.

## From performativity to environmental performativity

In his book *How To Do Things With Words*, the philosopher J.L. Austin (1962) showed how words do more than just describe. They set in motion chains of events. They have *impacts*. To make the case, he parses out the three different components of a speech act: the semantic meaning of the utterance, the intention behind the making of the utterance, and the eventual effects of the utterance. Austin describes the things that need to be in place for an utterance to *do something*. He refers to these as felicity conditions. They determine the relationship between an utterance's various components. In their presence, intentions and utterances lead to desired outcomes. They are performative in the sense that something was *done* by their being said. Austin showed how poorly language operates as a purely representational force. Instead, speakers, listeners, and referents inhabit a causally connected world.

Judith Butler used the performativity concept to understand the societal impacts of gender categories. Building on Foucault's anti-essentialism – that the identity of a thing is not an essential, timeless, inalienable aspect of it – Butler stresses that a body's gender is not pregiven. Instead, it is stabilised through the ‘stylized repetition of acts through time’ (Butler 1988, 521), through which bodies become materialised as specific gender identities. Gender is performative in that it constitutes ‘the identity it is purported to be’ (Butler 1990, 24). The ‘front against positivism and essentialism’ (Licoppe 2010, 187) in Butler's writing on gender is the same as the front Austin mounts against linguistic representationalism. Namely, that things are not out there in the world waiting to be described. They are constituted by sayings and doings.

STS scholars have extended Austin's and Butler's ideas to analyse the performativity of scientific and economic statements. In *The Embeddedness of Economic Markets in Economics*, Callon (1998b) describes how the reality of ‘the market’ and ‘the economy’ are brought into being by economists. He argues that ‘economics, in the broad sense of the term, performs, shapes and formats the economy, rather than observing how it functions’ (Callon 1998b, 2). The ‘calculative devices’ (Latour 1987) employed in techno-scientific and economic knowledge production are particularly important actors. As Callon explains, they ‘do not merely record a reality independent of themselves; they contribute powerfully to shaping, simply by measuring it, the reality that they measure’ (1998b, 23).

In the Actor Network Theory (ANT) approach Callon helped form, the term *network* is used to emphasise the constellations of people, materials, semeiotic devices, and calculative tools that are needed to make, do, or say something (Callon 1990; Law 1992). The term allows for the agency of nonhuman actors in creating performative effects. Where Butler's work grappled with the ways that socio-cultural performances (of discrete gender categories) are materialised, the focus on hybrid networks made greater concessions to the world-making impacts of a more diverse cast of nonhuman actors and materialities (Callon 2007, 2009b).

This move towards a more materially engaged theorisation of performativity has been taken further by new materialist and post-humanist scholars, most notably Karen Barad. Barad uses the term ‘cuts’ to refer to the way material configurations of an observer and their ‘apparatus of observation’ (2003, 815) carve out objects from the world. The reiteration of these cuts is necessary because the boundaries they create forever creak under the weight of complex material connectivity (Barad 2007, 2011). Making such cuts is far from an exclusively human endeavour. To combat the anthropogenic hubris which Barad detected in much social science writing on performativity, Barads develops the performativity concept to accommodate the way diverse material forms (including nonhuman life) differentiate themselves from their environments (Barad 2003, 2011).

We offer this brief genealogy of performativity to situate our paper within its wider theoretical arc. Where Barad developed an account of performativity in relation to what they saw as Butler's (and other's) excessive focus on socio-linguistic forces, we are now mindful of the critiques being levelled at post-humanist writing (of which Barad's is emblematic) and its excessive focus on the performativity and agency of matter. The concern is that such an emphasis on a ‘flattened’ materialist ontology collapses the consequences, actions, and agencies of diverse more-than-human actors into an undifferentiable ethical form (Rekret 2016). Some scholars, especially those working in the eco-Marxist tradition, have suggested that in the pursuit of a less speciesist account of agency, post-humanists are inattentive to the unique impacts human activity have had in driving socio-ecological collapse, and the uneven way the harms of anthropogenically driven environmental change are distributed (Hornborg 2016; Malm and Hornborg 2014).

We offer our environmental performativity concept in a way that accommodates the contributions new materialists have made in conceptualising the agency and mattering of nonhuman life whilst responding to the criticisms made about the depoliticising tendencies of their writing. Like other scholars writing to this brief (Giraud 2019; Lemke 2021), our intention is to grapple with the unique material and political impacts of anthropogenic activity without denying the agential and vibrant ways that nonhumans exert their influence and make their worlds. As a result, our review intentionally focusses on the ways that human representations and governance frameworks materialise new natures in specific ways. Yet we are also interested in the ways that various material forces inform, shape, disrupt, complicate, or overflow their anthropogenic representation. Across the three constituent components of the environmental performativity-whole (object making, environment making, regime making), we show how different material, semiotic, discursive, cultural, and calculative forms exert their world-making influence. To help capture this dynamic, we use the term *performance* in relation to the process through things are made, and *performative* in relation to those makings.

It is in this spirit that we review and categorise the explosion of scholarship from the mid-2000s onwards that has sought to understand the energy and finance being ploughed into environmental governance in performativity terms. It is to this environmental performativity scholarship – and the three types of performativity we read into the literature – that the paper now turns. To help build a unified conceptual framework for other scholars to engage with, we include in our review a small amount of scholarship that does not present its findings in ‘performativity’ terms but which can nevertheless be understood as having performative implications. This writing can be understood within, and will help elucidate, our conceptual framework.

## Object making

**Figure 1.**
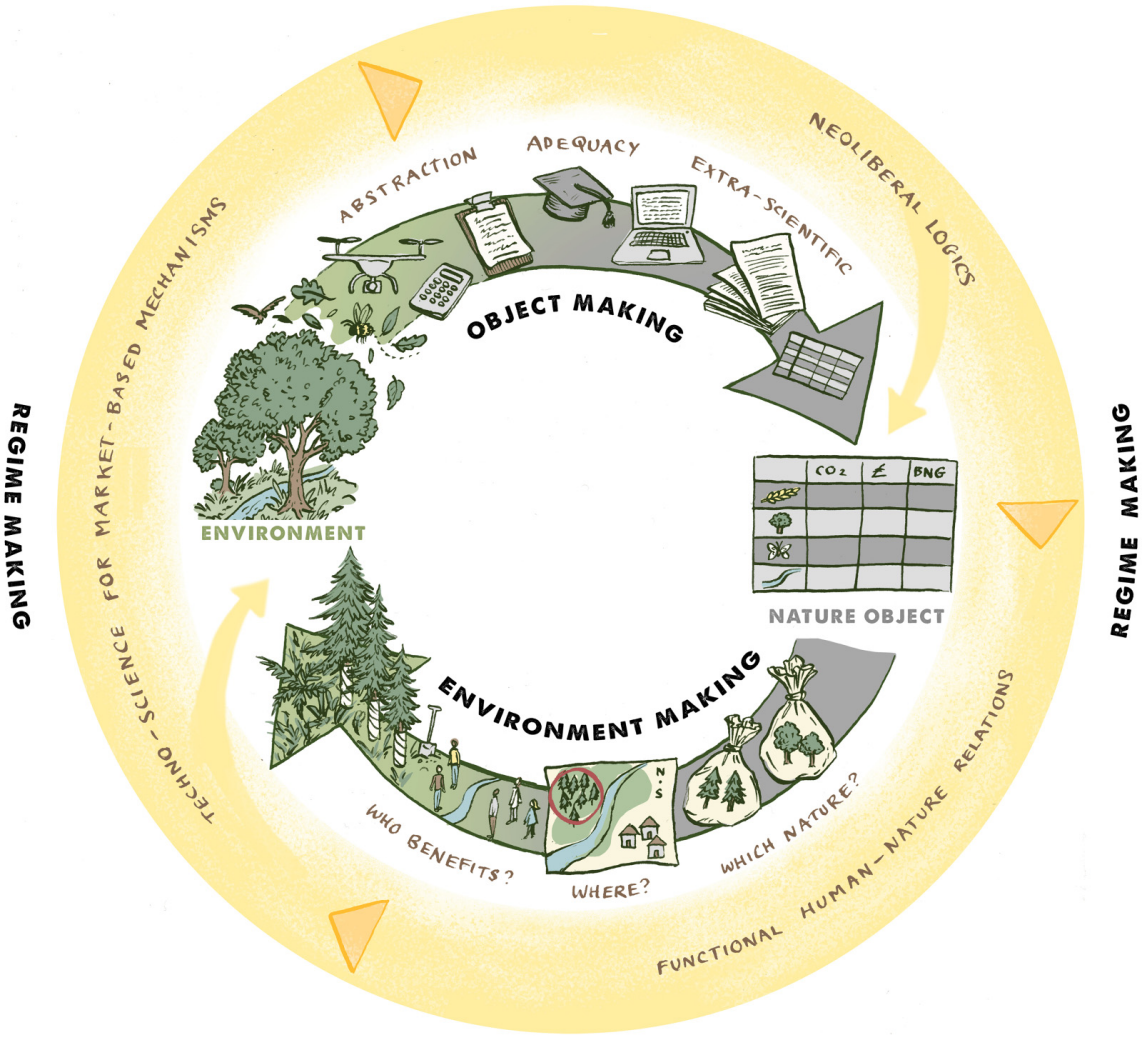
Environmental performativity. The image was produced in collaboration with artist and environmental communicator Vivien Martineau.

What exactly is being made in governance schemes that *make natures*? As the brochure would have it, it is trees, soils, habitats, riverside buffer strips, inert stored carbon, and so on. Whilst such materialities are indeed created, it is the representations of those things – ‘discrete noun-chunks of reality’ (Castree 2003, 280) – that the schemes must also deal in. They get stabilised from the messy world and inserted into inventories, spreadsheets, and databases (Latour 2005, 1987; Lorimer 2008). Such ‘translations’ rely on the sustained performances of assemblages of human and nonhuman actors, and calculative and materialsemiotic devices (Latour 2004; Law 2009). Following the lead of political ecologist Morgan Robertson, we term this process *object making*: the minting of durable, portable, bounded, enumerable things from messy and multiple natures. We refer to the things being made as *nature objects*: a translation that can be seen in the arrow moving from left to right in Figure 1*.* We describe the three aspects of this process: abstraction, adequacy, and extra-scientific.

### Abstraction

Calculative and empirical technologies are the main props of the object making performance (Gabrys 2020; Gabrys et al. 2022). Depending on the *making natures* scheme in question, specific ecological qualities are identified as being salient and desirable; selections which facilitate the completion of primary data capture and secondary calculations (Eden 2012; Robertson, Lave and Doyle 2023a). There is a distinct spatiality at work (Robertson, Lave and Doyle 2023b) in the equations that perform these abstractions (Lohmann 2014). GIS technologies, photographs, ecological surveys, spreadsheets, and fencing infrastructures all demarcate the natures that falls *outside* the project perimeter and thus the calculable spaces within which abstractions, enumerations, and representations can take place (Lansing 2012). Technologies – like drones – which assist in the surveillance and observation are particularly important devices in generating these abstractions (Millner et al. 2024). They allow an environmental resource to be abstracted into a nature object that can be transported offsite allowing it to be sold, traded, and governed elsewhere (Robertson 2012; Robertson, Lave and Doyle 2023a). This is a society preoccupied with auditing and accounting turning its attentions to environmental management (Bebbington and Larrinaga 2024).

Metrics have a particularly important role to play in these nature object abstractions (McElwee 2017). They provide common units through which heterogeneous and remote environments can be expressed in commensurate terms (Cooper 2015). In no-net loss biodiversity projects, for example, metrics allow the ecological value of an ecosystem in one location to be compared and replaced with one of ‘equal’ value (Robertson 2000). These metrics can ‘see’ no net loss, even across spatially remote and materially heterogeneous ecologies (Carver 2023). Such metrological containment can become sites of tension between nature's material tendency towards indeterminacy and queerness (Barad 2011) and an anthropogenic organisational impulse towards fixity and enumeration. Whilst the nature objects that get minted through specific performances might be internally coherent, they are always at risk of being ‘overflowed’ (Callon 1998a) by the ‘uncooperative’ (Bakker 2004) materialities on which they are predicated (Bakker 2012). This tension can sometimes undermine the viability of the metrics for those actors who are charged with translating complex ecosystems into stable numbers, figures, and stats (Hatanaka and Konefal 2024).

Many of those contributing to the environmental performativity programme invoke the ANT notion of *translation* to make sense of this phenomenon (Callon 1984; Law 1992). Translation refers to the way complex multi-actor assemblages, calculations, and negotiations are represented by a single thing that can be transported and brought to other actors for new rounds negotiation and calculation. These translations hold together networks of actors, materialities, and technologies that underpin markets and subsidy schemes for environmental services (Doganova and Karnøe 2015). The forest is assembled with ecologists, spreadsheets, and quadrats to be translated into a representation of its carbon content; a representation that can then be circulated amongst new actors (say a carbon broker) and calculations (the emissions budgets of the offset purchaser) (Carton, Lund and Dooley 2021). Less *all the world's a stage*, more *everything in it is an actor.*

### Adequacy

The above process of abstraction exposes the nature object to a trial of legitimacy. Shorn from the location and technologies involved in its production, nature objects must continually be framed in ways to convince external stakeholders of their adequacy. One can only know whether the performance is adequate if relevant actors consent to the object's entry into the circuits of exchange (Robertson 2012). This might happen when’s a nature object gets authorised through an accreditation standard, when a land manager gets remunerated through a subsidy scheme, or when a project receives finance through a sustainable investment mechanism.

There are no hard and fast rules for what constitutes adequacy. As a result, actors are strategic and reflexive about their performances and the fragility of the representations they uphold (Cusworth et al. 2023; Lippert 2015). They repeat their performances (take new photos, make new measurements, etc.) to insist on the link the nature object has to a specific material reality (Lansing 2012). And they make a show of selecting calculative tools that are regarded as being best able to describe a given ecology (Nost 2015). Particularly for inchoate technologies, speculative displays of environmental recovery are deployed to overcome the hesitancy the might otherwise be slowing their adoption (Price, Morris and Morris 2024).

What is needed for some performance to be adequate is brought into most clear relief when the performance ‘misfires’ (Callon 2010). Misfires are those interventions that fail to have the desired or anticipated performative effects. They tell of the brittleness of the performative process and the constant investments that are needed to make the performance stick. They speak to the difficulty in satisfying Austin's ‘felicity conditions’ (Austin 1962). Beunza and Ferraro's (2019) and Blok's (2011) studies, which show the upkeep involved in keeping the performance believable, are instructive. In their financial sector cases studies, steps were taken by officials after their scheme failed to attract commercial interest. These studies tell of the ‘performative texture’ (Licoppe 2010) of a situation that defines when some gesture, utterance, or calculation succeeds in creating something convincing. Performative texture varies from case to case, as do the adjustments actors make in their bid to generate the required performative outcomes. Whilst a translation can for a time be successful, it requires constant improvisation (Callon and Law 2005).

Adequacy is a cultural measure, not a material one. Many studies describe examples of *making natures* schemes that are adequate in the sense that actors give their assent they representations they trade in, but that deliver little in the way of environmental change. If substantial gaps should open up between representation and reality – which is highly likely given the indeterminacies inherent to measuring ecological processes (Robertson 2006) – then schemes that perform ecological change may, in fact, be materially impotent or create perverse ecological outcomes (Price, Morris and Morris 2024).

Carbon accountants, for example, who do so much to stabilise unruly emissions into tradeable and governable nature objects, can pass around, lose, or externalise emissions onto other actors in a value chain (Lippert 2015). Measurement technologies can, to take another example, allow for more natural capital stored within a landscape to be ‘known’, even when new natures are not being ‘grown’ (Stanley 2024). In examples of more deliberate deception, selective performances can disappear environmental risks and other damages to suit the needs of scheme officials (Mathews 2014). In macro-economic terms, the Green Economy has performatively created a ‘virtual’ world of environmental investments (Bracking 2015; Hook and Laing 2022) and ‘phantom credits’ (Greenfield 2023) that are unmoored from actual environmental recovery. At their worst, object making performances can simulate de-carbonisation and environmental recovery without materialising actual change, deterring more radical and meaningful mitigation ((Markusson, McLaren and Tyfield 2018). In theoretical terms, this is when the semioticcultural aspect of environmental performativity becomes most clearly uncoupled from its material counterpart; an uncoupling that does not, from the perspective of invested parties, necessarily make the representation *inadequate*.

### Extra-scientific considerations

In the bid for adequacy, ‘good science’ only gets you so far (Nadaï, Cassen and Lecocq 2023). Cultural and social considerations of issues that appear to be exclusively scientific must be made. Castree (2020) refers to these as ‘extra-scientific’ considerations. Compliance with voluntary standards (Valiergue and Ehrenstein 2023) or strategic allegiances with NGOs, for example, help reassure relevant parties of the normative merit and calculative honesty of the object making performance. Such multi-actor and international castings (between NGOs, state actors, scientific institutions etc.) make a show of the fact that appropriate tools are being brought to bear on transnational environmental issues with justice-relevant outcomes. Algorithmic calculations become intermingled with socially rich ‘qualculations’ (Callon and Law 2005) to demonstrate adequacy as a comprehensive socio-technical achievement (Nadaï, Cassen and Lecocq 2023). Trust, transparency, and socio-political legitimacy emerge, here, as major determinants in the performative power of environmental governance interventions (Ghosh 2024; Ransmayr 2023). Shows of calculative conservatism (Stanley 2024) and the precautionary principle (Robertson, Lave and Doyle 2023a) – manifest in formulaic modifiers that undercount rather than oversell the natures being made – help perform the laudable intentions of scheme officials. Here, it is not only the scientific rigour that counts; an overt political commitment to trust and transparency also helps to convince other parties of the adequacy of a translation.

The growing move to integrate social values into the design of environmental governance programmes represents another extra-scientific bid for adequacy. Over the latter half of the 2010s, the Intergovernmental Science-Policy Platform on Biodiversity and Ecosystem Services (IPBES) included ‘nature's contribution to people’ and the importance of non-Western knowledge in their governance systems (McElwee et al. 2020). Here, the impulse to technocratically measure and manage is extended to the delivery of social values in environmental governance (Tadaki and Sinner 2014). Doing so allows environmental and justice considerations to be endowed with ‘the values of objectivity and legitimacy associated with quantitative finance’ and other ‘objective’ disciplines (Beunza and Ferraro 2019, 533). As we discuss more below, however, such performances can reinforce the power imbalances that they were designed to redress (Turnhout et al. 2020) and make public resource users themselves into data points (Eden 2012).

Particularly in commercial settings, actors promise the delivery of ostensibly non-capitalist goals (e.g. wildness, traditionality, justice, Indigenous knowledge) to enhance their appeal (Cusworth et al. 2022). In emission offset markets, these promises enhance the ‘virtuous’ appeal (Paterson and Stripple 2014) of ‘charismatic carbon’ (Wang and Corson 2015). The pursuit of good optics can sometimes also lead to the loss of information, rather than an expansion in the quality and quantity of the ecological data being captured. In Lehmann's (2019) study of cookstove carbon offsetting projects, for example, complex outcomes are overlooked in the reporting process due to the way they are seen to dilute the legitimacy – and thus the value – of the negative emission credits being generated.

Some of the steps taken to ensure credibility and adequacy can be surprising. In his study of payment for ecosystem service schemes, Ghosh (2019) describes the ‘appetite for imprecision’ in the software that calculates payment rates for farmers delivering agri-environmental outcomes. It is not so much the pursuit of more and more accurate calculative devices that actors are drawn to. Rather, it is the devices that can be used by a diverse range of actors in the field, and those that produce the sort of representations amenable to the *making natures* scheme in question. Lave, Doyle and Robertson (2010) reach a similar conclusion. In their study of stream restoration projects, preference for up-to-date ecological methods is trumped by the need for metrics that can be rolled out as a standardised package for the diverse actors that are networked together in the scheme, and for metrics that produce representations most amenable for the market-based mechanisms for which they are destined (Lave, Doyle and Robertson 2010). The scientific thirst for precision is always proscribed by practical and economic pressures (Robertson 2006).

In sum, as an object of study and governance, natures are stabilised through repeat performances. Scientists, technicians, and calculative devices – that stabilise, bound, and enumerate – are cast in the lead role. Depending on the performative texture of the situation and the strategies deployed by relevant actors, translations are then made adequate and compelling through displays of accountability, rigour, repeatability, standardisation, calculative conservativism, transparency, imprecision, scalability, accessibility, calculative opacity, and association with morally upright actors like NGOs or other independent bodies.

But what happens once these nature objects are loosed on the world? What performative effects do *they* have?

## Environment making

The performances which create nature objects also (re)shape the material world. They have performative outcomes. Fences are erected, trees are planted, rivers are re-wiggled. Over the course of this section, we refer to this form of performativity as *environment making*. We show how the natures and actors that are most amenable to the object making process benefit from whatever making natures scheme predominates. This culminates in a cyclical process of object making and environment making. The material world formats the nature objects that are minted through the performances we describe above, whilst those objects go on to shape the material world in a process we describe below. This can be seen in Figure 1 in the arrow going from nature object back to environment. The representation of this dynamic as a cycle is not to say that every real-world-making natures scheme features recurring interactions between the two performative processes. It is, instead, a heuristic to suggest that that each *can* inform the other in impactful ways. Although the reshaping of environments is clearly the point of environmental governance programmes, we show that the process is loaded with justice-relevant implications for the more-than-human worlds they implicate. This is because the environment making performance determines *which* natures are made, *where* they are made, and *who benefits*.

### Which natures?

Environmental historians, political ecologists, and geographers have highlighted that in modern Euro-American environmental governance, the pursuit of legibility has long been a priority. Natures have been made mappable, extractable, and controllable from distant locations (Scott 1998). This has, in turn, shaped which environments are materially produced through regimes of environmental regulation and commodification. Typically, environments are produced in standardised forms to make them easily commensurable and accountable (Barua 2023). This ‘plantation logic’ involves streamlining, ordering, and accelerating nonhuman life into financially productive ecologies (Chao 2022).

This plantation logic is now being grafted onto new pro-environmental rootstock. As ecosystems get cast as nature-based solutions to socio-ecological problems, they are being approached as a technology that can be optimised for the delivery of environmental gains (Markusson 2022). The process is always geared around the creation of natures that perform well in whatever ecological or public good metric underpins the scheme in question (Carton and Andersson 2017). In a political and economic atmosphere preoccupied with carbon *sequestration* rather than *storage*, for example, young and quick growing trees are more desirable than older and slower growing ones (Palmer 2021). As a result, juvenile trees are planted before old ones receive protection. The object making process described in the above section is, in other words, profoundly performative in the way it materialises specific natures.

As a result, schemes that *make natures* tend to produce neat, simple, and orderly environments, geared around the production of species that yield good results for the markets and subsidy programmes for which they are being cultivated (Kellokumpu 2022; Lyons and Westoby, 2014a). By contrast, messy and diverse ecosystems like scrubland and naturally regenerating treescapes, whose benefits are harder to measure, lose out (Stanley 2024). The phenomenon has other-than-ecological consequences. Rich, situated, and spiritual relationships between humans and the more-than-human world are often inscrutable to measurement, and so are liable to fall off the governance radar (Lave, Doyle and Robertson 2010; Zografos and Robbins 2020). For financial instruments like ESG investment services, socio-ecological issues whose impacts stand to be felt across the whole economy (like climate change and deforestation) have traction in a way that issues (like animal welfare) that are confined to a single sector do not (Brice et al. 2022).

In contemporary environmental governance contexts, where data-intensive methods are seen to be vital to the informed governance of complex ecological problems, black-box software has a powerful yet inscrutable environment making performativity (Kloppenburg et al. 2022; Nost 2022). This is because of the way they process ecological data, map out policy priorities, assign subsidies, and calculate payment rates. There are justice concerns with delegating so much decision-making power to such tools that are, at once, opaque, epistemically authoritative, and materially productive (Nost and Goldstein 2022).

In sum, if there are natures that benefit from their legibility to capital and techno-scientific measurement, then natures which fail to be adequately abstracted also fail to be materialised through the mechanisms that promise to realise nature recovery (Robertson 2006). Highly particular translational processes yield very specific environments. They define *which natures* are made.

### Which natures, where?

The object making process defines not just *which* natures are made, but *where.* Mapping software creates spatial fixes when trading off between environmental and economic objectives. Cities that are most important for a state's economic revenue, for example, benefit from the environment making performativity of environmental protections (such as flood defences), irrespective of the environmental or social externalities those protections may produce elsewhere in the region (Gray 2021). The location of the natures produced through environmental governance programmes bear the ‘signature’ (Lave et al. 2014) of the software(s) that handle spatially sensitive ecological data. In some instances, these performative signatures can be detected in unexpected socio-economic metrics like house prices (Bowden et al. 2021).

Labels and maps have powerful effects in shaping the material worlds they describe. For example, land cover designations drive the spatiality of the environment making performative process. An area of forest labelled ‘wilderness’ might be granted conservation status whilst an area of the same forest mapped as a ‘commercial frontier’ might be logged (Braun 2002). This, in turn, triggers different environmental controls, shaping where in the world environments are created and where they can be razed. In his classic study of land use designation, Robbins describes the power relations that determine how landscape photographs help fix the land cover types of given areas, and how those decisions shape where governance interventions flow (2001). In this way, visualisations of environmental resources are, at once, instantiations, articulations, and reproducers of power (Gabrys et al. 2022). Technologies like drones and satellite surveillance systems which offer streams of visual and spatial data about the resources/territories being governed are reconfiguring the geographies of environmental governance (Millner et al. 2024).

Given the preponderance of Global South producers and Global North consumers of environmental credits, the *which* and *where* of environment making can take on a neocolonialist hue (Hook and Laing 2022; Lyons and Westoby 2014a). The economic opportunity manifest in carbon markets is, for example, catalysing a series of land grabs in the Global South, erasing traditional knowledge systems in the process (Navarro 2022; Richards and Lyons 2016). Carton and Edstedt (2021) use the term ‘sacrificial zones’ to describe the areas in which complex ecological dynamics and more-than-human relations are replaced by natures that can most expediently produce sought after environmental benefits like carbon sequestration. Nature objects, such as the maps used to specify certain sections of forest as carbon, can legitimise the restoration of one area and the destruction of another (Braun 2002; Lansing 2012). Here, well-documented dynamics of uneven economic development are playing out through the delivery of environmental recovery programmes.

### Who benefits?

Clearly the point around *which natures* materialise, and questions of *where,* leads to the more salient point of *who benefits*. The calculative devices used to translate environments into governable nature objects are imprinted with the power structures that define how *making natures* schemes are designed; an impression that carries over to the environments the schemes go on to materialise (Bakker and Ritts 2018; Nost and Goldstein 2022). As Edstedt and Carton (2018) show in their study of a tree planting scheme in Uganda, consumers and project officials in the Global North specify which social benefits get pursued, rather than the communities proximate to the tree planting activity. This tends to mean that the social or ecological goals pursued by offset purchasers are prioritised ahead of locally preferred outcomes (Wang and Corson 2015). Although there are always multiple ways of being with the more-than-human world, schemes that codify and streamline those relations do a violence to those whose knowledge and desires do not ‘make the cut’ (Barad 2011).

Although environmental market-based mechanisms can offer meaningful developmental avenues for countries in the Global South, critical social scientists remain unconvinced by their radical and redistributive potential (Osborne 2015). The extension of the profit motive into environmental realms means that the commodification process can lead to the unequal distribution of the economic gains that are generated from using nature as a resource (Buscher and Fletcher 2020). The ‘uneven geographies’ of carbon sequestration, for example, are marked by global power imbalances (Carton and Edstedt 2021). These schemes enable polluting nations and companies (typically in the Global North) to continue their emissions trajectories by displacing the legal and commercial pressure to de-carbonisation their value chains. Lyons and Westoby use the terms ‘carbon colonialism’ (2014a) and ‘carbon violence’ (2014b) to describe the way that offsetting markets are upending land values culminating in exploitative labour arrangements, socio-ecological simplification, and land grabs. The problem is not just a North-South one. Within offset producing regions in the Global South, power dynamics between government officials, local communities, and commercial operators dictate how the benefits and harms of *making natures* schemes are divvied up. Larger landowners are, for example, better equipped to participate in the new Green Economy than smaller landowners with more precarious forms of tenure (Lansing 2014).

The process of environment making performativity dictates *which* natures are made, *where* they are made, and thus *who benefits* from their creation: three processes with profound justice implications.

## Regime making

Schemes that make natures do more than just mint governable nature objects and materialise environments. They shape how environmental issues are problematised, how governance frameworks are designed, and how human–nature relations are imagined. We refer to these outcomes as *regime making*. These performative outcomes, which exceed the intentions and jurisdictions of any single scheme, unfold at a higher level of abstraction relative to the other two performative processes. They shape how environmental problems are understood and approached at a more ideological level. These higher order conditions feed back into the lower level processes of environment- and object- making described above. The regime making phrase nods to Wilshusen's conclusion that economistic and techno-scientific approaches to environmental finance are ‘performative above and beyond the… work that they may or may not achieve.’ (Wilshusen 2019, 2).

With so much environmental governance obtaining to a neoliberal programme, the scholarship we present below shows how the neoliberal ideals that are fed into *making natures* schemes are reproduced as they are (re)articulated. These relate to the preference for techno-scientific knowledge production, market-based mechanisms, and functional human–nature relations. In Figure 1, this regime making process is represented by the circular arrow towards the outside of the diagram. It whips around the environment-object making cycle, informing how programmes of environmental management are designed and which natures get materialised. It picks up speed as its ideologies, problem-definitions, and solutions are reiterated over time.

### Techno-science for market-based mechanisms

Lansing's (2012) study of the verification of a carbon offsetting project offers an empirical entry-point to witness how the performances of object making and environment making have second-order regime making impacts of their own. He documents the ecological data capture and reporting performances that forest-level bureaucrats undertake to sustain the credibility of the carbon offset credits being generated. Whilst the legitimacy conjured up through these performances is, on the face of it, about imbuing nature objects (credits, figures, datasets) with the adequacy they require to enter into the circuits of exchange, there is a second performance underway. The constant investments made to index grounded materiality to abstract representation ‘is critical for the performative process where both the object of exchange, *and the frame of exchange itself*, become simultaneously emergent’ (Lansing 2012, 212, emphasis added). The adequacy of the abstraction and the legitimacy of the frame that allows for their sale emerge interdependently. They are, in his words, ‘coeval’.

These dynamics are indivisible from the neoliberal paradigm in which they emerge (Robertson 2012). Here, the idea that market-based mechanisms should occupy a central role in navigating environmental collapse is performed by the actors and methods who help to produce the representations those market-based mechanisms trade in. The impacts of this regime making performativity are cumulative. As more time, money, and effort is invested into market-oriented approaches to environmental problems, that framing gets regarded as the inevitable way to do environmental governance (Markusson, McLaren and Tyfield 2018; Nost 2015). The concern from critical commentators is that such market-friendly mechanisms and techno-scientific methods legitimise and perpetuate the same extractive human–nature relations that catalysed environmental collapse in the first place (Lohmann 2014; Sullivan 2018). As Sullivan (2018) prompts us to ask in her study of environmental investment initiatives, what remedies for environmental deterioration are made possible when ecological health is measured in economistic terms?

In this neoliberal regime, the political and scientific are far from distinct realms. Dominant discourses around the expedience of extending market relations into environmental governance regimes have formatted the goals, funding distribution, and interests of the research sector (Robertson 2006). The presence of the profit motive also unevenly distributes the extent to which different stakeholders in a scheme are allowed to contribute to its design and knowledge production (Carton 2020). This defers deliberative political discourse around the desirability of more radical approaches to environmental governance (Swyngedouw 2011). The exclusive power science is assumed to wield in terms of creating scalable and effective solutions is performative in the way it creates, legitimises, and reproduces its own ideological footing (Machen 2018; Machen and Nost 2021).

Esther Turnhout, Neves and de Lijster (2014) use the term ‘measurementality’ to refer to the way techno-scientific forms of knowledge production are privileged in environmental governance spheres. Prevailing political preference for market-friendly interventions (e.g. offsetting schemes) shape how scientific knowledge production is employed within environmental policymaking. They diagnose ‘a process of articulation in which ecological phenomena are made legible to specific political and economic logics… the environment is measured and represented in a very specific way: one that enables a specific mode of governing that happens to be associated with very specific sets of scientific, political, or economic interests’ (2014, 581). This regime making performativity is problematic not just because of what it privileges, but what it forecloses. Whilst the pursuit of specific forms of ecological science makes the integration of biodiversity governance into market-based mechanism easier, it leads to ‘an impoverishment of the biodiversity research agenda… [and] an impoverished understanding of biodiversity itself’ (Turnhout, Neves and de Lijster 2014, 581).

### Functional human–nature relations

Metaphysical and ethical questions about more-than-human relations also get performatively formatted by the way environmental issues are framed. In the ecosystem service model, one of the mainstays of contemporary neoliberal governance, humans are cast as homogenous users of amenities provided by a monolithic Nature (Sullivan 2017). This singularises diverse ways of being with the more-than-human world, and it marginalises the place of non-Western and Indigenous cosmologies in the process (Flint et al. 2013; Stoeckl et al. 2021). In Gibbs’ (2010) study of Australian water resource management, for example, the prominence of Eurocentric understandings of water cycles has undermined the perceived legitimacy of Aboriginal modes of interacting with water, even though they are more sensitive to local conditions. The stylised implementation of the ecosystem service schemes performs and amplifies singular human–nature relations (Kolinjivadi et al. 2019; Kull, Arnauld de Sartre and Castro-Larrañaga 2015).

There has been some public pushback to the functional way environmental governance programmes perform human–nature relations. Groups like IPBES are beginning to fold in non-Western cosmologies into the way they conduct research and broker the science–policy interface (McElwee et al. 2020). This is being done to move towards a more democratised and pluralistic approach to knowledge production and stakeholder consultation (Borie et al. 2021). Critics, however, remain sceptical. Firstly, about the capacity for economistic methods to properly represent the spiritual dimensions of human–nature relations, and secondly about the way that the performative display of progressive politics may defer critique of the powerknowledge constellations on which they are built (Schaafsma et al. 2023; Sullivan 2018; Tadaki and Sinner 2014).

Such concerns are being exacerbated by the susceptibility of democratisation and pluralisation discourses to corporate capture. With actors in voluntary offset schemes markets leveraging the social benefits and emancipatory potential of their wares (Paterson and Stripple 2014; Wang and Corson 2015), capital is proving itself endlessly creative in the way it is seeks to perform its ability to mutate into a ‘civilised market’ that is attentive to the social dimensions of environmental change (Callon 2009a). Here, economies for environmental services and carbon offsets are assumed to be ‘good’ simply because they trade in biological goods (Asdal et al. 2023). Regime making performativity, here, amounts to a symbolic politics whereby viewing publics are reassured that appropriate and proactive steps are being taken to safeguard the future of the environment (Blatter 2009; Carton et al. 2023). At its worst, though, this performance amounts to strategic window dressing and greenwash (Blühdorn and Deflorian 2019): simulations of de-carbonisation and environmental recovery that rarely tally with their supposed real-world referents (Beck and Oomen 2021; Blühdorn 2007). Here, the performance of environmental recovery (where the performance term is closer to a folk definition meaning ‘hollow’ or ‘insincere’) helps performatively sustain the neoliberal regime.

Schemes that *make natures* tend to perpetuate the ideals that are fed into them: they sediment mercenary and singular ways of relating to the more-than-human world; they present political decisions to solve environmental crises via the extension of market-friendly logics as apolitical, inevitable, and expedient; they privilege techno-scientific methods of knowing and governing. Given the hegemonic position it occupies in environmental governance contexts, the neoliberal regime is (re)made through the object and environment making performances in a way that legitimates its own epistemological, political, and ontological foundations (Lansing 2012; Nost 2015). This amounts to Escher's staircase of self-legitimisation: a closed loop of performativity that seals off criticism by setting the terms by which it can be judged. Speaking in relation to the ecosystem service model, Kolinjivadi and colleagues describe this as a sort of blindness: ‘We are unable to “see” the neoliberal governmentality pervasive within the payment for ecosystem service construct because we can only explore new possibilities from the unconscious acceptance of the cultural construction itself’ (2019, 8). Breaking out of this bind, specifically through reflexive and strategic disruption, is where the paper turns next.

## Conclusion: Reflexive performativity

What lessons might be drawn from all this scholarship? First is a general scepticism towards market-based mechanisms, techno-scientific fixes, subsidy programmes, and offset markets in terms of their ability to address ecological collapse and climate breakdown. We also suggest, though, that there is also a less diagnostic, more emancipatory message on offer. Latent within the object making, environment making, and regime making processes that constitute the environmental performativity framework, there is a lesson about contingency and disruption. In this concluding section, we articulate how the anti-determinism and anti-essentialism that are inherent to environmental performativity – that natures are constantly (re)made as they are articulated – is an invitation to cast new actors and write new scripts. Given their interrelations, making an intervention that affects one part of the environmental performativity-whole can have ripple effects on other elements. Through careful and reflexive engagement, therefore, dominant frames and incumbent ways of managing nature can be disrupted, destabilised, and ameliorated.

Building on the term developed by Cusworth et al. (2023) we refer to the strategic engagement with the contingency and influence of environmental governance design as *reflexive performativity.* It speaks to the merit of tracing how arcane metrological and scientific frameworks shape how and where new natures get materialised, how overarching societal agendas reiterate their social and technological foundations, and how experimentation with alternative modes of governance can realise desired worlds. Whilst their original paper highlights the dangers that exist when powerful and unscrupulous actors try to shape the metrological worlds they inhabit to their advantage, we also see the hope that is latent in the analysis. If there are multiple ways that environmental management schemes can be designed and delivered, and if those various paths have the power to create better or worse worlds, then choices can be made with more just environmental futures in mind. Here, the value of our disambiguated set of performativity analytics comes into clearest focus. Armed with a clear understanding of how environments, representations, and ideology interact, these reflexive engagements can be strategic and calculated.

Critical environmental social science scholarship offers insight into the performative disruptions that might help fulfil these possibilities. Scholars working in the field of digital ecologies have shown how digital technologies can create novel affordances for more carefilled interspecies relations (Searle, Turnbull and Adams 2023; Turnbull et al. 2023). Sensing, recording, and other filmic practices can similarly offer glimpses into alternative experiences and unseen multispecies relations that might otherwise be excluded in traditional governance settings (Hunter and Searle 2024; Turnbull and Searle 2022; Westerlaken et al. 2023). Technologies such as drones can, under the right circumstances, further facilitate the democratisation of conservation governance (Gabrys 2019; Millner et al. 2024), whilst technologically mediated participation in the design of environmental governance can open up opportunities for Indigenous, rural, and marginalised communities to take control of their own environmental data (Millner 2020). More generally, holistic, relational, and integrative ecological metrics can materialise environments that are less predicated on the attainment of very singular ecological goals (Stanley 2024). In the terms we offer in this paper, this scholarship shows that when abstracting environment into governable nature objects, new technologies can create environments with better outcomes for the more-than-human actors they implicate.

Given the interactions between the different elements that constitute environmental performativity, a reflexively designed disruptive intervention in one domain can have knock-on effects on the others. Just as the enactment of neoliberal politics substantiates neoliberalism's ideological foundations (Kolinjivadi et al. 2019), the deployment of environmental governance interventions adhering to alternative socio-political principles can confer legitimacy on alternative socio-political regimes. Growing interest in the notion of soil *health* rather than soil *fertility* is, for example, beginning to galvanise broader industry interest in soil as a vibrant relational materiality (Krzywoszynska 2019). This is, in turn, spurring greater investments in ecological research and extension services orientated towards more ambitious agroecological goals (Cusworth and Lorimer 2024). Similarly, NGO efforts to map national emissions budgets according to a ‘fair per capita share’ – which are nominally about creating a more fair global economy for emissions trading – are working to turn the emissions market itself into a site of contestation (Blok 2011). Alternative modes of measurement can substantiate regimes that endorse more just forms of more-than-human biopolitics and environmental governance (Wakefield and Braun 2019).

These opportunities raise important questions for the role of social science scholarship. Is the job to create these alternative measures and metrics? Or is it to nurture the sort of societal ‘felicity conditions’ that are needed to allow them to take root? Whilst we cannot offer answers to those questions here, our hope is that the environmental performativity concept can help illuminate some potential avenues of inquiry and action: to resist the temptation to abandon modernist ‘commitments to the ideals of progress, truth, and improvement’ (Lorimer 2020, 228) and to reflexively repurpose those commitments with more radical ends in mind. Scholars and social actors must, in this spirit, learn to ‘scavenge’ (Robertson, Lave and Doyle 2023a) or ‘hack’ (Chandler 2018) the technologies of modernity to create metrological frameworks that can performatively generate more just and ecologically comprehensive programmes of environmental governance.

Whilst many of the studies we have reviewed in the paper speak to the pathos of environmental governance, in highlighting both the power and the contingency of their performances and performative effects, they also offer a sliver of hope. Namely that social scientists and activists need to be alert to the way the object, environment, and regime making performances unfold, and the way their performative outcomes interact. And so, whilst commercial actors and scheme bureaucrats are already reflexive about the performativity of environmental governance design (Cusworth et al. 2023; Lansing 2012), those interested in making alternative futures also need to get in on the act.
